# Applicability of magnetic seeds for target lymph node biopsy after neoadjuvant chemotherapy in initially node-positive breast cancer patients: data from the AXSANA study

**DOI:** 10.1007/s10549-023-07100-0

**Published:** 2023-09-08

**Authors:** Steffi Hartmann, Maggie Banys-Paluchowski, Elmar Stickeler, Jana de Boniface, Oreste Davide Gentilini, Michalis Kontos, Stephan Seitz, Gabriele Kaltenecker, Fredrik Wärnberg, Linda Holmstrand Zetterlund, Hans-Christian Kolberg, Sarah Fröhlich, Thorsten Kühn

**Affiliations:** 1 Department of Obstetrics and Gynecology, University Hospital Rostock, Rostock, Germany; 2 Department of Obstetrics and Gynecology, University Hospital Schleswig-Holstein Campus Lübeck, Lübeck, Germany; 3 Department of Obstetrics and Gynecology, University Hospital Aachen, Aachen, Germany; 4 Department of Surgery, Capio St. Göran’s Hospital, Stockholm, Sweden; 5 Department of Molecular Medicine and Surgery, Karolinska Institutet, Stockholm, Sweden; 6 San Raffaele Hospital Milan, Milan, Italy; 7 Laiko University Hospital, Athens, Greece; 8 Department of Obstetrics and Gynecology, University Medical Center Regensburg, Regensburg, Germany; 9 Department of Obstetrics and Gynecology, City Hospital Karlsruhe, Karlsruhe, Germany; 10 Department of Surgery, Sahlgrenska Academy at University of Gothenburg, Gothenburg, Sweden; 11 Department of Obstetrics and Gynecology, Marienhospital, Bottrop, Germany; 12 Department of Obstetrics and Gynecology, Die Filderklinik, Filderstadt, Germany; 13 Department of Obstetrics and Gynecology, University Hospital Ulm, Ulm, Germany

**Keywords:** Breast cancer, Neoadjuvant chemotherapy, Target lymph node, Targeted axillary dissection, Magnetic seed

## Abstract

**Purpose:**

Currently, various techniques are available to mark and selectively remove initially suspicious axillary lymph nodes (target lymph nodes, TLNs) in breast cancer patients receiving neoadjuvant chemotherapy (NACT). To date, limited data are available on whether the use of magnetic seeds (MS) is suitable for localizing TLNs. This study aimed to investigate the feasibility of MS in patients undergoing target lymph node biopsy (TLNB) or targeted axillary dissection (TAD) after NACT.

**Methods:**

Prospective data from the ongoing multicentric AXSANA study were extracted from selected patients in whom the TLN had been marked with an MS before NACT and who were enrolled from June 2020 to June 2023. The endpoints of the analysis were the detection rate, the rate of lost markers, and the potential impairment on magnetic resonance imaging (MRI) assessment.

**Results:**

In 187 patients from 27 study sites in seven countries, MS were placed into the TLN before NACT. In 151 of these, post-NACT surgery had been completed at the time of analysis. In 146 patients (96.0%), a TLN could successfully be detected. In three patients, the seed was removed but no lymphoid tissue was detected on histopathology. The rate of lost markers was 1.2% (2 out of 164 MS). In 15 out of 151 patients (9.9%), MRI assessment was reported to be compromised by MS placement.

**Conclusion:**

MS show excellent applicability for TLNB/TAD when inserted before NACT with a high DR and a low rate of lost markers. Axillary MS can impair MRI assessment of the breast.

**Trial registration number:**

NCT04373655 (date of registration May 4, 2020).

## Introduction

Currently, ongoing clinical trials compare different surgical axillary staging procedures for breast cancer patients who receive neoadjuvant chemotherapy (NACT) and achieve conversion from initially clinically node-positive (cN+) to clinically node-negative disease (ycN0). Various international guidelines recommend either sentinel lymph node biopsy (SLNB) alone or targeted axillary dissection (TAD) as less radical alternatives than axillary lymph node dissection (ALND) [1], which represented the gold standard for decades. In TAD, the largest biopsy-confirmed axillary metastasis or most suspicious axillary lymph node, determined as the target lymph node (TLN), is marked before NACT and removed at post-NACT surgery along with the sentinel lymph node (SLN) [2]. This procedure reliably reduces the false negative rate (FNR) from 13% for SLNB alone to 5% [3]. To allow selective removal of the TLN after NACT, any pre-NACT marking technique must permit its reliable identification after NACT even in cases of complete clinical or radiological response in the axilla. Available techniques include visual identification of a TLN tattooed by carbon particles [4], image-guided preoperative wire insertion after pre-NACT placement of metallic clips [5, 6], or probe-based techniques using radioactive iodine seeds [7, 8], radiofrequency identification tags (RFIDs) [9], radar markers [10, 11], or magnetic seeds (MS) [12]. While the use of carbon particles is cheap and reliable, this technique may be associated with more extensive surgical dissection to visualize the TLN [4]. The placement of metallic markers and subsequent image-guided wire localization after NACT yields detection rates (DR) of only 70–78%. This technique requires high expertise for the secondary localization procedure and is less reproducible in clinical routine [5, 6]. Furthermore, it is associated with logistic challenges (localization on the same day as surgery is scheduled) and discomfort for the patient. In addition, the wire can dislocate between localization and surgery or when positioning and prepping the patient in the theater. In contrast, probe-guided techniques appear promising since the above-described additional localization procedures can be avoided and the workflow thus improved. As an example, the prospective multicenter RISAS trial tested the use of radioactive iodine seeds which were identified by a gamma probe and reported a DR of 94.1% [8]. Radioactive seeds are, however, not allowed in many countries due to radiation protection regulations [1].

In the localization of non-palpable breast lesions, the placement of MS with subsequent probe-guided tumor resection has shown non-inferiority compared to the most popular and widely used technique of stereotactic clip localization [13]. An MS is a 0.9 × 5 mm stainless steel seed and is inserted into the lymph node via an 18 Gauge needle usually under ultrasound guidance. It is transcutaneously detectable up to a penetration depth of 30 mm [12]. The necessary magnetometer probe generates an alternating magnetic field to transiently magnetize the iron-containing seed [14]. Although MS are considered magnetic resonance imaging (MRI) compatible, they cause extinction artifacts of 4–6 cm on breast MRI [12]. Since 2020, MS are approved to reside in soft tissue without a time limit, which allows their insertion into the TLN before NACT [15]. In the few available studies that assessed the feasibility of marking the TLN with an MS, high DRs up to 100% could be shown (Table 1). In most patients, however, the TLN was first marked with a metallic clip pre-NACT, and the MS was only placed after NACT to avoid wire localization [16–27]. This two-stage procedure does, however, not overcome the problem of ultrasound-guided clip detection in patients with a good response to NACT in the TLN.

**Table 1:** Studies reporting feasibility of axillary lymph nodes marking with magnetic seeds

Study	Design	Number of patients with marked lymph nodes (*n*)	Number of magnetic seeds applied before NACT (*n*/%)	DR of TLN/magnetic seed (%)
Barry 2023 [16]	Prospective, unicentric	221	54	NR/100
Martínez 2022 [17]	Prospective, multicentric	81	44	100/100
Miller 2021 [18]	Prospective, unicentric	134	0	NR/94.1
Simons 2021[19]	Prospective, unicentric	50	0	100/100
Reitsamer 2021 [20]	NR	40	2	100/100
Mariscal Martínez 2021 [21]	Prospective, unicentric	29	0	100/100
McCamley 2021 [22]	Prospective, multicentric	6	1	100/100
Žatecký 2021 [23]	Retrospective, multicentric	7	0	85.7/100
Laws 2020 [24]	Retrospective, unicentric	12	0	75.0/100
Greenwood 2019 [25]	Retrospective, unicentric	35	0	NR/97
García-Moreno 2019 [26]	Case report	1	1	100/100
Rodriguez Gallo 2018 [27]	Case report	5	0	NR/NR

*DR* detection rate, *NACT* neoadjuvant chemotherapy, *TLN* target lymph node, *NR* not reported

The objectives of the current study were to determine the DR and the rate of lost MS after TLN marking before NACT in the largest so far investigated study cohort, as well as to investigate how often the assessment of an MRI to determine tumor response under NACT was limited by the MS in the axilla.

## Patients and methods

### AXSANA study

The ongoing AXSANA study (NCT04373655) is an international, multicenter, prospective registry study initiated by the European Breast Cancer Research Association of Surgical Trialists (EUBREAST) that started recruitment in June 2020. Breast cancer patients with cN+ disease who receive at least four cycles of NACT are eligible. Axillary staging is performed according to institutional and national standards and includes ALND, SLNB, target lymph node biopsy (TLNB), or TAD (i.e., SLNB + TLNB). Co-primary endpoints are invasive disease-free survival, axillary recurrence rate, health-related quality of life, and arm morbidity for the different surgical staging procedures. The trial has high-quality standards with 100% of the datasets being monitored by breast surgeons. In patients scheduled for TLNB or TAD, any currently available techniques for marking the TLN are allowed [28].

A secondary endpoint of the trial is the performance of different marking techniques for the TLN.

### Patients

The current analysis selectively included patients enrolled in the AXSANA study who had TLN marking with an MS (Magseed^®^, Endomagnetics Ltd, Cambridge, United Kingdom) before NACT and who had undergone axillary surgery by June 01, 2023. The number of labeled TLNs was not prescribed in the study protocol. At surgery, the TLN was identified using the SentiMag^®^ (Endomagnetics Ltd, Cambridge, United Kingdom) handheld magnetometer probe. Clinical lymph node status before and after NACT was assessed according to institutional standards (palpation ± imaging of choice). Pathological complete response (pCR) was defined as the absence of invasive tumor cells in the breast and axilla [29]. Thus, the presence of isolated tumor cells in any axillary lymph nodes (ypN0i+) was defined as non-pCR.

### Statistical analysis

For descriptive analysis, absolute frequencies and proportions were reported for categorial parameters and median values (minimum–maximum) for quantitative parameters. DR was defined as the proportion of patients with successful perioperative identification of at least one lymph node marked with an MS out of all included patients. The rate of lost markers was determined from the proportion of all unsuccessfully removed magnetic seeds out of all initially inserted MS. Statistical analysis was performed using SPSS® version 27 (IBM, Armonk, New York, USA).

## Results

**Fig. 1:**
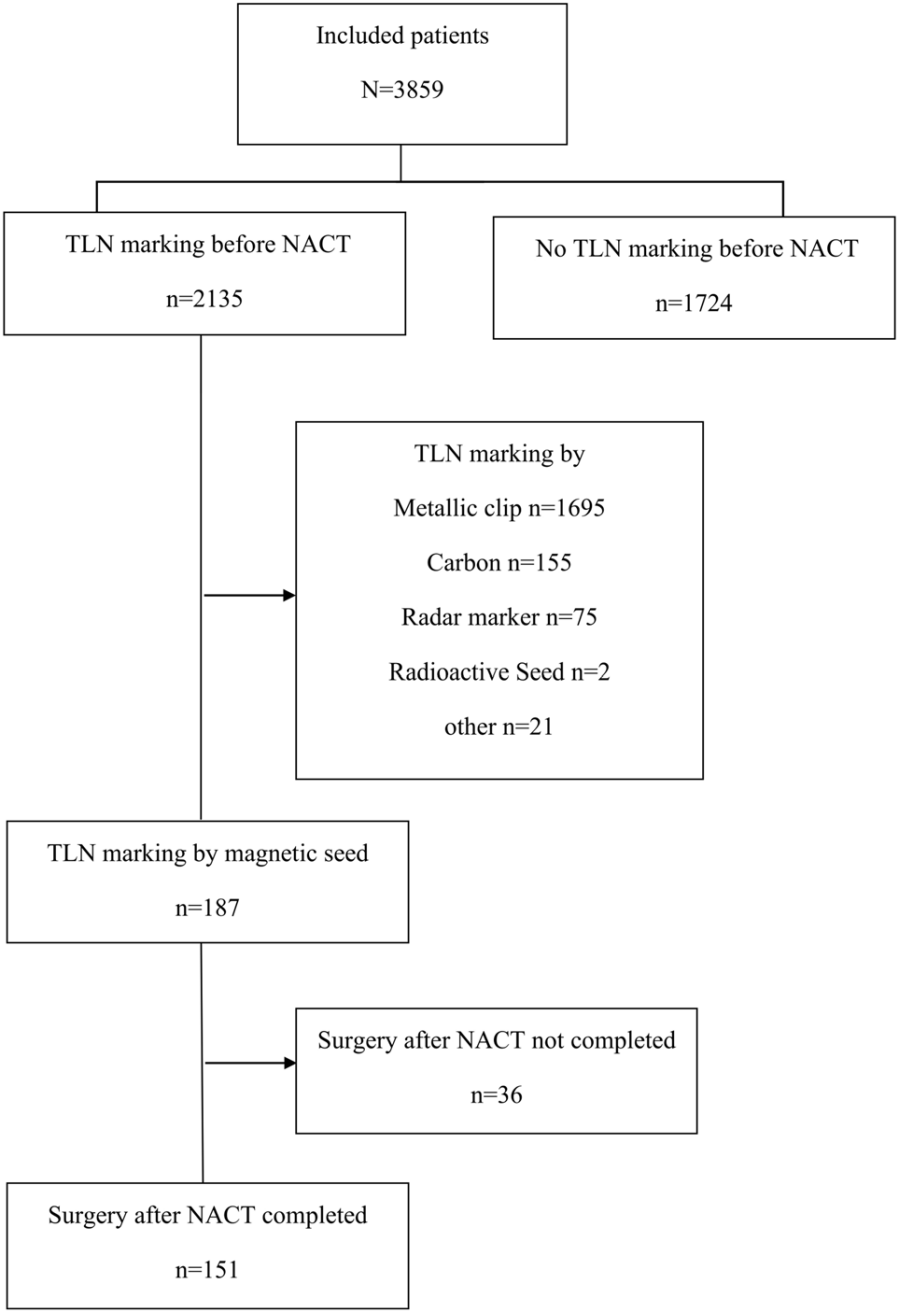
Flowchart of AXSANA study cohort included until June 01, 2023

During the here studied time frame, 3859 patients from 27 countries and 286 study sites were included in the AXSANA study. In 2135 out of 3859 patients (55.3%), the TLN was labeled before NACT, and amongst these, MS was inserted in 187 cases (8.8%). All 151 out of these 187 patients (80.7%) in whom surgery had been performed and documented by June 01, 2023, were included in the current analysis (Fig. 1). These patients were recruited from 27 study sites in seven countries. Clinicopathologic characteristics are shown in Table 2.

**Table 2:** Baseline characteristics (*n* = 151)

	*n* (%)
**Age** (years)^a^	51 (24–82)
**Body mass index** (kg/m^2^)^a^	25(18–44)
**Clinical tumor stage at diagnosis**	
cT1	38 (25.2)
cT2	88 (58.3)
cT3	23 (15.2)
cT4	2 (1.3)
**Number of suspicious lymph nodes before NACT**	
1–3	135 (89.4)
≥ 4	15 (9.9)
Missing	1 (0.7)
**Histopathological tumor type**	
Ductal	135 (89.4)
Lobular	9 (6.0)
Mixed ductal and lobular	2 (1.3)
Other	5 (3.3)
**Tumor subtype**	
HR+HER2−	66 (43.7)
HR+HER2+	33 (21.9)
HR-HER2+	18 (11.9)
HR-HER2-	34 (22.5)
**Proliferation Ki67** (%)^a^	40(5–98)
**Tumor multicentricity**	
Yes	24 (15.9)
No	127 (84.1)
**Clinical lymph node status after NACT**	
ycN0	107 (70.9)
ycN+	44 (29.1)
**Type of breast surgery**	
Breast-conserving surgery	97 (64.2)
Mastectomy	53 (35.1)
Missing	1 (0.7)
**Pathological lymph node status after NACT**	
ypN0	77 (51.0)
ypN0i+	2 (1.3)
ypN1mi	7 (4.6)
ypN1	49 (32.5)
ypN2	13 (8.6)
ypN3	2 (1.3)
Missing	1 (0.7)
**Pathological complete response after NACT**	
Yes	60 (39.7)
No	90 (59.6)
Missing	1 (0.7)

*NACT* neoadjuvant chemotherapy, *HR* hormone receptor, *HER2* human epidermal growth factor receptor 2; ^a^ median (minimum–maximum)

In all patients, MS were placed before NACT and were the only markers used for TLN labeling. In 138 (91.4%) patients, only one MS was inserted, and in 13 (8.6%) patients, two were used, resulting in a total of 164 MS applied. The median time between TLN labeling and TLN removal was 168 (58–291) days. In 150 (99.3%) patients, TLN marking was performed under ultrasound guidance.

At least one MS-marked TLN could be detected in 146 out of 151 patients (DR 96.0%). The median number of histologically detected TLNs was 1 (0–6). In three out of six patients with unsuccessful TLN identification, the MS had been successfully removed, but no lymph node was histologically detectable in the surgical specimen. In one patient a probe-guided TLN detection had not been attempted since the patient underwent primary ALND; in two further patients, the MS had been detected by the probe in the ALND specimen but the TLN had not been evaluated separately by the pathologist.

A TAD was planned in 127 out of 151 patients (84.1%). The median number of TAD nodes removed was 2 (0–9). The SLN was labeled before surgery by a magnetic tracer in 51 patients (40.2%), technetium in 50 patients (39.4%), blue dye in four patients (3.1%), indocyanine green in 1 patient (0.8%), and dual marking in 21 patients (16.5%). The SLN could be detected in 116 out of 127 cases (91.3%) and corresponded to the TLN in 80 (69.0%) patients. ALND was performed in 74 patients (49.0%), in 55 patients (74.3%) within primary surgery, and in 19 patients (25.6%) as a secondary procedure.

Of the 164 initially applied seeds, 162 (98.8%) could successfully be removed after NACT. The rate of lost markers is thus 1.2%. In one patient, only one of the two initially applied MS was detectable; in the second case, it remained unclear whether the MS was still in situ because no intraoperative localization was performed. The seed was not mentioned in the pathology report. In both patients, no additional imaging was ordered to verify the *in-situ* retention of the seeds.

In 121 patients (80.1%), information on experience with the MS technique for TLN labeling was provided by the study site: in 43 cases (35.5%), at least 30 TLN labelings with MS had been performed at the sites to date. None of the patients with non-detection of TLN or lost marker had undergone surgery at such a high-experience site.

**Fig. 2:**
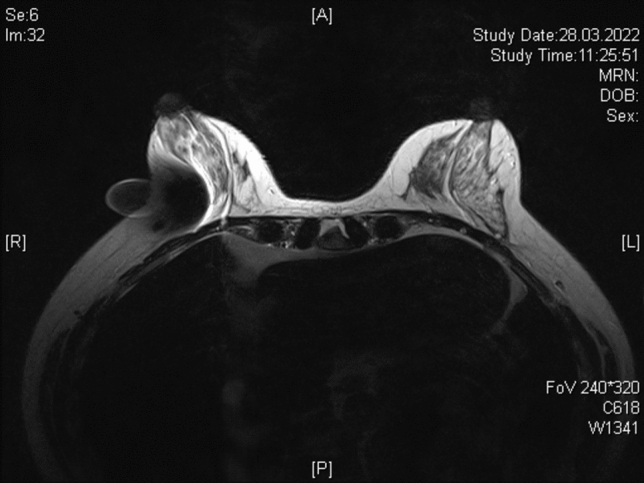
Impairment of breast MRI assessment due to an artifact caused by an axillary magnetic seed in craniolaterally located cancer of the right breast

A breast MRI was performed in 31/151 patients after NACT but before surgery (20.5%). Radiologists described MRI artifacts caused by MS in 28/31 patients (90.3%). In 15 of these (48.4%) the radiologist described an impairment of the interpretation of the MRI due to artifacts caused by the axillary magnetic marker (Fig. 2) resulting in a rate of 9.9% (15/151 patients) for the entire study cohort.

## Discussion

For patients who initially present with a clinically positive lymph node status and convert to ycN0 through NACT, the historic gold standard of ALND is increasingly abandoned in favor of less invasive techniques such as SLNB, TLNB, or the combination of both (TAD). Although long-term data on the oncologic outcome of these procedures are still scarce, most available studies, which still have insufficient statistical power, indicate no impairment of regional recurrences or disease-free survival [30–32]. Large prospective multicenter studies like AXSANA and MINIMAX (NCT04486495) will provide clarification.

TAD significantly lowers the FNR of SLNB alone and is currently the most popular procedure to stage the axilla in this patient cohort as could be shown in an international survey published recently by the EUBREAST network [33].

A recent meta-analysis compared the DR for different TLN marking techniques, such as iodine seeds (95.6%), metallic clips (91.7%), and carbon particles (97.1%). The identification of metallic clips, the currently most popular procedure, is associated with highly variable results (DR 70–98%) [34]. In the German SenTa study, a prospective trial using metallic clips for TLN labeling, the DR for the TLN was only 77.8% in a multicenter setting [5]. This suggests an unfavorable reproducibility of ultrasound-guided wire localization of axillary clips with a potential impact on experience, the type of clip, and whether or not intraoperative ultrasound was used.

High DRs have been described for the use of carbon particles. No comparative data are currently available from clinical trials as to whether this non-probe, visual-only detection of TLN results in more extensive dissection in the axilla or increased postoperative morbidity [4].

The DR for MS-marked TLNs reported here (96.0%) outperforms the recently published DR for radioactive seed-marked TLNs (94.1%) in the multicentric prospective RISAS study [8], which is also a probe-guided technique. In contrast to iodine seeds, MS are approved for long-term residence in the body and are not subject to legal regulations based on radioactivity.

The use of MS, as a wireless and non-radioactive procedure appears as an attractive alternative to avoid disadvantages of the established techniques. This procedure has shown excellent results for the localization of non-palpable breast lesions [35]. Since limited data are available on their use in TLNB, they were not included in the above-mentioned meta-analysis regarding different techniques for TLN marking [34]. The restricted approval of MS to remain in the body for up to 30 days was removed in 2020 [27]. Therefore, in most studies published to date, the number of patients with MS placed before NACT is very low (Table 1). Mainly, the TLN was marked before NACT using a metallic clip and the MS was placed in the TLN after NACT under ultrasound guidance [18, 19, 21, 23–25, 27]. Since initially clip-marked TLNs cannot be visualized by ultrasound after NACT in about 20% of patients [5], a targeted application of the seed after NACT under ultrasound guidance in these cases seems not feasible. In addition, MS appear to be significantly more likely to dislocate from the TLN into the perinodal tissue when inserted after NACT as compared to an application before NACT (27% versus 1.7%, *p*<0.001) [16]. To our knowledge, we present here the largest prospective cohort analyzing the performance of MS placed before neoadjuvant treatment. In this setting, any additional localization procedure before NACT can be avoided. Our data show a high DR of 96.0% for TLNs MS-marked before NACT and confirm preliminary data from smaller series [16, 17, 20, 22, 26].

The fact that all cases with unsuccessful localization procedures in our study occurred within the learning curve of the respective site suggests that even better results can be expected in experienced hands. Clip and carbon labeling studies of TLN have also described higher DR with increasing expertise of the center with each technique [5, 36]. Thus, for the SenTa study, a lower DR (69.3%) was described for sites with little experience (less than 20 cases) with clip marking of the TLN than for sites with at least 20 cases (DR 88.6%) [5]. Overall, however, the DR was significantly lower than that determined here for MS. Although two-thirds of the patients underwent surgery after MS labeling in this study at sites with limited experience (less than 30 MS applications), there was a high DR and only two lost seeds, so the procedure can be assumed to be straightforward to learn.

A potential disadvantage of magnetic markers compared to other techniques is an impairment of MRI assessment, and thus the monitoring of tumor response under NACT [35]. The current study is the first in which the impairment of breast MRI assessment by axillary-applied MS was investigated. Although the MS was located in the axilla and not in the breast, the assessability of tumor response in the breast after NACT was impaired in half of the patients in whom breast MRI was performed preoperatively. Therefore, in patients in whom breast MRI is to be performed to assess tumor response, a different marker should be considered to label the TLN.

Although the chance of unsuccessful seed removal is low (1.2% lost marker), patients should be informed about this potential risk. In particular, if MRI is indicated during follow-up.

In addition to a possible impairment of the assessability of a breast MRI, the high cost of the MS and the acquisition of the probe system, especially compared to metallic clips and carbon suspension, may limit their application in clinical practice [37].

A strength of the present analysis is the large number of patients enrolled in a prospective, multicenter study design. All datasets have been monitored by surgeons experienced in the field of breast surgery. A limitation is that no direct comparison with competing techniques is possible so far. This relates especially to innovative probe-guided procedures.

## Conclusion

TLN marking using MS is a highly effective method that outperforms most competing techniques. As a wireless and non-radioactive procedure, this technique appears highly attractive in the setting of TAD. However, it should be noted with planned breast MRI that artifacts due to axillary MS may limit its assessability.

## Data Availability

The dataset generated and analyzed during the current study is available from the corresponding author upon reasonable request.
